# Idiopathic retroperitoneal fibrosis causing unilateral ureteral and sigmoid colon obstruction: A case report

**DOI:** 10.1097/MD.0000000000006105

**Published:** 2017-02-17

**Authors:** Ting Yan, Yujuan Wang, Zhijun Liu, Xiaolei Zhang, Qian Wu, Mingrong Xi

**Affiliations:** a Department of Gynecology and Obstetrics, West China Second University Hospital, Sichuan University, Chengdu, Sichuan; b Department of Gynecology; c Department of Pathology, Guizhou Provincial People's Hospital, Guiyang, Guizhou, China.

**Keywords:** idiopathic retroperitoneal fibrosis, sigmoid colon obstruction, ureteral obstruction

## Abstract

**Objective:**

The present report aimed to present a unique case of idiopathic retroperitoneal fibrosis (RPF) presenting features of unilateral ureteral and sigmoid colon obstruction. RPF is a rare disorder with unclear etiology.

**Case report:**

A 43-year-old female had a 10-day history of lower right abdominal and lumbar pain. Gynecological examination, ultrasound, and computed tomography (CT) were all suggestive of right ovarian tumor. An enhanced CT showed right-sided hydronephrosis. The patient was diagnosed as having ovarian cancer. Ten days after hospitalization, a right intraureteral stent with a double-J catheter was inserted. Upon exploring the abdomen, unyielding RPF was encountered. A partial sigmoidectomy and colostomy were performed. Postoperative pathological results suggested idiopathic RPF. She received steroid treatments.

**Conclusion:**

RPF is a rare disease that can be misdiagnosed. Our understanding about its presentation has to be improved and it should be considered as a differential diagnosis for patients presenting with abdominal diseases.

## 1 Introduction

Retroperitoneal fibrosis (RPF) is an uncommon condition with an estimated incidence of 1.38 cases per 100,000 people.^[1,2]^ Its etiology and pathogenesis are unclear.^[2–4]^ It can be classified as idiopathic and secondary.^[2,5]^ It was first described in 1905 by the French urologist Albarran and became fully described as an entity in 1948 by Ormond. RPF is a chronic inflammatory process of the retroperitoneum characterized by the presence of fibro-sclerotic tissues involving retroperitoneal structures.^[6]^ RPF has an insidious clinical onset characterized by flank, back, or abdominal pain, and constitutional symptoms such as malaise, fever, anorexia, and weight loss.^[3]^ Laboratory finding may include elevated erythrocyte sedimentation rate, elevated C-reactive protein, elevated urea and creatinine levels, anemia, polyclonal hypergammaglobulinemia, elevated alkaline phosphatase, and presence of antinuclear antibodies.^[4,7,8]^ Urine examination is usually normal, but may reveal microscopic hematuria or pyuria.^[4,7,8]^ Ultrasound usually reveals extensive, well-defined, hypoechoic mass accompanied with various degree of hydronephrosis.^[2,9]^ Computed tomography (CT) usually reveals a plaque that is isodense with muscle.^[2,10]^ Magnetic resonance imaging can be useful, but differentiation between benign and malignant fibrosis may be difficult.^[2,11]^ Positron emission tomography can also be useful.^[2]^

The present study aimed to report a unique case of idiopathic RPF presenting with features of unilateral ureteral and sigmoid colon obstruction, and clinical workup suggestive of an ovarian cancer.

## 2 Case report

A 43-year-old female presented with a 10-day history of abdominal and lumbar pain accompanied by nausea, vomiting, and constipation (1 bowel movement per 2–3 days). She denied oliguria, dysuria, and hematuria. She had a history of right adnexa benign cyst surgery more than 5 years ago. Unfortunately, she could not provide her precise medical history. She specifically denied previous exposure to ergot, methysergide, or β-blockers.

Physical examination showed stable vital signs. Her abdomen was soft and flat with normal bowel sounds. No pain sensation was detected in the bilateral renal area, and the superficial lymph nodes were not palpable. Gynecological examination showed a solid and fixed mass (9 × 9 cm) in the right rear uterus. The anus examination showed a solid enclosed mass attached to the rectum and no blood on the fingerstall.

Laboratory tests showed white blood cell count of 14,790 cells/mm^3^ (normal 3500–9500 cells/mm^3^), hemoglobin of 9.1 mg/dL (normal 11.5–15.0 mg/dL), and eosinophil cell count of 2200 cells/mm^3^ (normal 20–520 cells/mm^3^). Blood urea nitrogen and creatinine were normal. The erythrocyte sedimentation rate (ESR) was 64 mm/h (normal, 0–30 mm/h) and C-reactive protein was 127.08 mg/L (normal, 0–5 mg/L). CA125 was 313.4 U/mL (normal, 0–35 U/mL) and CA199 was 87.04 U/mL (normal, 0–39 U/mL). Urine routine examination showed microscopic hematuria.

**Figure 1:**
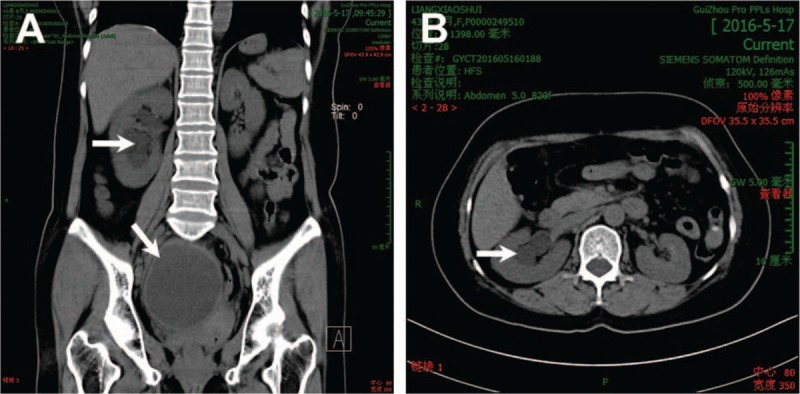
Computed tomography (CT) findings of idiopathic retroperitoneal fibrosis. CT showing soft-tissue low-density mass surrounding the right adnexa and right-sided hydronephrosis. CT = computed tomography.

**Figure 2:**
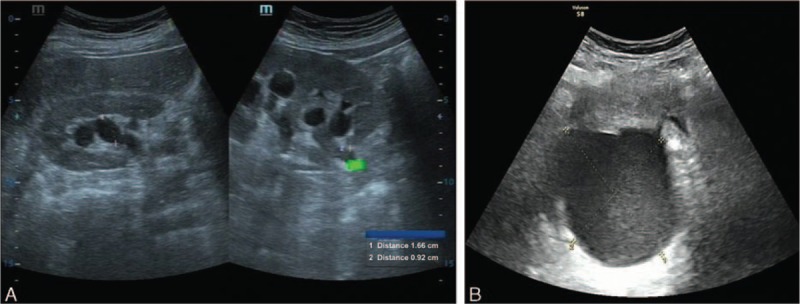
(A) Urinary ultrasound showing right-sided hydronephrosis and irregular upper section of the right ureter. (B) Gynecological ultrasound showing a cystic mass (8.8 × 7.5 × 8.6 cm) in the right rear uterus. The mass was grossly well circumscribed.

Contrast-enhanced computed tomography (CT) showed right-sided hydronephrosis and a low-density mass around the right adnexa. The imaging features strongly suggested an ovarian epithelial tumor (Fig. 1). Single-photon emission computed tomography (SPECT/CT) showed slight atrophy of the right kidney, right-sided hydronephrosis, right kidney blood perfusion, mild damage to the glomerular filtration function, and irregular upper urinary tract. Ultrasound showed a cystic mass (8.8 × 7.5 × 8.6 cm^3^) in the right rear uterus, which was considered as an ovarian tumor (Fig. 2A). Abdominal ultrasound was normal. Urinary system ultrasound showed right-sided hydronephrosis and dilated upper section of right ureter (Fig. 2B). Ultrasound of the left kidney was normal. Gastrointestinal endoscopy showed level II chronic superficial gastritis. She was diagnosed with class I primary hypertension (low risk) using a 24-h blood pressure monitoring.

After a series of examinations and discussion, this patient was diagnosed with ovarian cancer, right-sided hydronephrosis, mild anemia, level II chronic superficial gastritis, and class I primary hypertension. Because the main treatment of ovarian cancer is surgery and the preoperative examination suggested right ureteral obstruction, a preoperative cystoscopy and exploratory laparotomy were performed.

Ten days after hospitalization, she was brought to the operating room. A preoperative cystoscopy was performed and a right intraureteral stent with a double-J catheter was inserted. Then, an exploratory laparotomy was performed via a midline incision. Upon entering the peritoneal cavity, slight bloody ascites and dense intra-abdominal adhesions were encountered in the right pelvic cavity. The classical glistening white, unyielding RPF was encountered. The plaque predominantly encased the pelvis, especially the right pelvis. The pelvic peritoneum showed thickening and edema, especially the retroperitoneum. The maximal thickness of the plaque was 1 cm. It formed a solid and fixed mass in the right lower pelvis. The omentum majus contractured and adhered densely to the anterior abdominal wall. The omentum majus densely encased the sigmoid colon and rectum. The sigmoid colon adhered to adjacent retroperitoneal structures densely and formed a stiff mass. The fixed sigmoid colon adhesion was 6-cm long with stiff wall, thickening and hardening of the intestinal wall, and obvious constricted lumen. The vermiform appendix was clearly visible and hard. The uterus was slightly expanded, with thickened serosa. The left adnexa was normal and the right adnexa could not be found. The Douglas pouch was closed because of dense adhesions. The right ureter was surrounded by dense fibrotic tissue with chronic inflammation and formed right ureteral obstruction. No abnormality was found when examining the liver, pancreas, spleen, stomach, small bowel, ascending colon, transverse colon, and descending colon. She underwent resection of the retroperitoneal mass, appendectomy, partial resection of the omentum majus, partial sigmoidectomy, proximal sigmoid colon fistula, and closure of the distal sigmoid colon. The patient recovered well after surgery. She was advised to go to the No. 1 Hospital of Peking University for steroid therapy.

**Figure 3:**
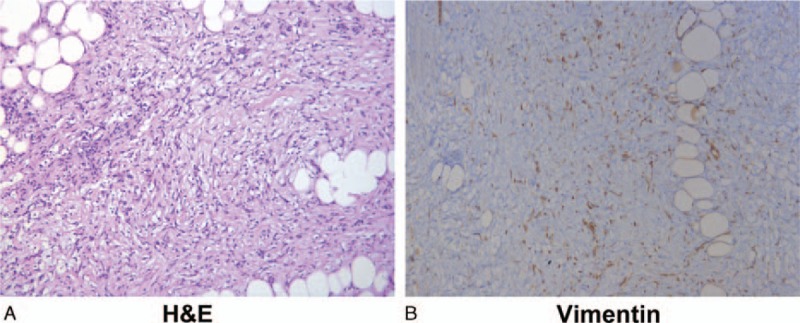
Idiopathic retroperitoneal fibrosis. (A) Photomicrograph from the surgical specimen using hematoxylin and eosin (H&E) (×200 magnification) showing mature fiber tissue mixed with fibroblasts, neutrophil granulocytes, plasma cells, and lymphocytes. (B) Immunohistochemistry for vimentin (×200 magnification) showing that the spindle stroma cells were positive for vimentin. H&E = hematoxylin and eosin.

The pathological diagnosis was idiopathic RPF. The fibrous component was interpreted as remarkable storiform fibrosis mixed with fibroblasts, neutrophil granulocytes, plasma cells, and lymphocytes (Fig. 3A). Immunohistochemistry showed that vimentin was positive (Fig. 3B), smooth muscle actin was partially positive, β-catenin was positive (cell membrane and cytoplasm), Ki-67 was very weakly positive (<1%), GIST-1 was equivocal, and desmin, CD30, anaplastic lymphoma kinase, CD117, CD34, α-inhibin, S-100, epithelial membrane antigen, and cytokeratin pan were all negative.

## 3 Discussion

RPF is a rare chronic inflammatory disease involving the retroperitoneum and causing compression of the retroperitoneal structures, especially the ureters, frequently leading to pyeloureterectasis.^[6]^ Several studies have reported about RPF, but the present report is probably the first to present idiopathic RPF with features of both unilateral ureteral and sigmoid colon obstruction.

The pathogenesis of idiopathic RPF is unclear. A number of hypotheses were made and include environmental factors, immunologic process (e.g., immunoglobulin G4-bearing plasma cell deposition), genetic factor, and local inflammatory complication of advanced atherosclerosis. There is an increased incidence of autoimmune disease and/or autoantibody positivity in idiopathic RPF and chronic periaortitis.^[1]^ However, some series revealed that associated autoimmune diseases and/or autoantibodies in RPF were not frequent.^[1]^ In this report, no immune system disease was diagnosed in the patient during hospitalization, but no relevant tests were performed at first since she had been initially diagnosed with ovarian cancer. The most common symptom of RPF is abdominal and back pain,^[12]^ which was present in this patient. This pain is typically dull and unchanged with posture and may radiate to the lower abdomen or groin.^[6]^ About 80% to 100% of patients with idiopathic RPF show ureteral involvement.^[12]^ Indeed, the patient reported here suffered from right-sided hydronephrosis.

There are no standard diagnostic criteria for RPF and routine laboratory tests are often consistent with those for inflammatory diseases. Levels of acute-phase reactants such as ESR and C-reactive protein are high in 80% to 100% of patients. These tests are often used to monitor the clinical course of this disease, but they do not necessarily mirror disease activity.^[13]^ Among all available examinations, ultrasound should be done as a first-line examination, especially in the presence of an azotemic patient, because it is inexpensive, reliable, and noninvasive. On ultrasound, idiopathic RPF appears as a hypoechoic or isoechoic mass that can involve the ureters. This mass could then explain the unilateral or bilateral hydronephrosis in these patients.^[2,13]^ Ultrasound of the patient in the present report showed a mass in the right pelvic cavity, right-sided hydronephrosis, and dilated upper section of right ureter.

Imaging studies such as CT and magnetic resonance imaging remain important in diagnosing RPF.^[14]^ They are noninvasive and usually provide a clear picture of the disease, which is typically that of a periaortic mass of soft-tissue density extending from the level of the arteries to the iliac vessels and frequently causing medial ureteral deviation and obstruction.^[3]^ The present report showed more specific right hydronephrosis using SPECT/CT.

We may distinguish 2 subtypes of RPF: idiopathic and secondary. A number of conditions may cause secondary RPF such as drug exposure, infection, surgery, and malignancy. After excluding these causes of secondary RPF, the diagnosis of idiopathic RPF is made, but nevertheless requires histopathological confirmation by biopsy.^[5]^ The patient in the present study had a history of surgery, but she could not be diagnosed with secondary RPF only based on this nonspecific factor. In some atypical cases, idiopathic RPF may be periduodenal, peripancreatic, pelvic, periureteral, or close to the renal hilum, all forms without aortic involvement. In these atypical forms, imaging usually reveals a poorly circumscribed retroperitoneal mass, making difficult the differential diagnosis and requiring a confirmation by biopsy.^[13]^ Therefore, despite being invasive, the histological examination of the lesion seen on imaging is still the most reliable diagnostic tool. Indeed, biopsy is the only modality that can rule out malignant, benign, or infectious etiologies.^[3]^ In the case reported here, hypertension was diagnosed using multiple blood pressure monitoring. Occurrence of hypertension was rarely reported previously.

There is no standard treatment for idiopathic RPF and it consists in a combination of medications and surgical intervention that is based on each physician's experience.^[14]^ The first-line of treatment for RPF is considered to be steroid therapy, regardless of the etiology of RPF.^[15]^ Since surgery itself may be a cause of RPF, surgery is usually kept to relieve obstructions. The patient in the present report was treated by right intraureteral stent insertion with a double-J catheter and diagnosed by surgery. It has been suggested that surgery can either be preceded or followed by steroid treatment.^[13]^ Our patient was a minority woman, and it was difficult for her to accept and continue a steroid treatment for up to 2 years. Because the optimal steroid management is not well described in the literature and because the authors of the present report did not feel confident in prescribing the steroid therapy, it was recommended that she consult to the No. 1 Hospital of Peking University for the subsequent therapy.

In conclusion, because of its rarity, diagnosis and differential diagnosis of RPF is difficult. Indeed, at presentation, the present case was highly suggestive of ovarian cancer in terms of symptoms, physical examination, and imaging. We should consider the possibility of RPF when patients present with localized symptoms such as flank, back or abdominal dull pain, and more especially when complicated with hydronephrosis. Elevated ESR, CRP, and antinuclear antibodies could also be used to suggest RPF. CT and MRI are the most reliable methods for the diagnosis of idiopathic RPF, and RPF should be considered in the presence of a soft-tissue mass surrounding the retroperitoneal structures and unexplained urinary obstruction.
