# Idiopathic retroperitoneal fibrosis causing unilateral ureteral and sigmoid colon obstruction: a case report: Erratum

**DOI:** 10.1097/MD.0000000000006529

**Published:** 2017-03-24

In the article “Idiopathic retroperitoneal fibrosis causing unilateral ureteral and sigmoid colon obstruction: a case report”,^[1]^ which appeared in Volume 96, Issue 7 of *Medicine*, Francesco Carubbi was credited as the article's only editor. In fact Francesco Carubbi and Shizhang Ling were both editors of this article.
